# Overlapping Cytomegalovirus Infection and Inflammatory Pouchitis After Liver Transplantation: When Antivirals Alone Are Insufficient

**DOI:** 10.1016/j.gastha.2026.100963

**Published:** 2026-04-10

**Authors:** Brody M. Fogleman, Ryan O’Leary, Tiffany Baker, Erin Forster

**Affiliations:** 1 Internal Medicine, Department of Medicine, Medical University of South Carolina, Charleston, South Carolina; 2 Inflammatory Bowel Disease, Department of Medicine, Medical University of South Carolina, Charleston, South Carolina; 3 Department of Pathology and Laboratory Medicine, College of Medicine, Medical University of South Carolina, Charleston, South Carolina; 4 Inflammatory Bowel Disease Center, Division of Gastroenterology and Hepatology, Department of Medicine, College of Medicine, Medical University of South Carolina, Charleston, South Carolina

**Keywords:** Cytomegalovirus (CMV) Pouchitis, Ileal Pouch-Anal Anastomosis (IPAA), Ulcerative Colitis, Secondary Pouchitis

## Abstract

Cytomegalovirus (CMV) is an infrequently reported etiology of secondary pouchitis in patients who have undergone total proctocolectomy with ileal pouch-anal anastomosis. We present the case of a patient with chronic antibiotic-dependent pouchitis, ulcerative pancolitis, primary sclerosing cholangitis, and orthotopic liver transplant who presented with hematochezia. Pouchoscopy showed ulcerated mucosa, and histopathology with immunohistochemistry confirmed CMV pouchitis. The patient was treated with antiviral therapy with improvement in bleeding and viremia. However, persistent refractory pouch inflammation ultimately required guselkumab. This case depicts the importance of considering CMV as a secondary etiology of pouchitis, especially in those with refractory or atypical features.

## Background

Total proctocolectomy (TPC) with ileal pouch-anal anastomosis (IPAA) is commonly performed in patients with medically refractory ulcerative colitis. Pouchitis is a common complication following IPAA.^1^^,^^2^ Most cases are idiopathic and respond to antibiotics, but 20%–30% of chronic pouchitis have secondary causes.^2^^,^^3^

Cytomegalovirus (CMV) infection is an infrequently reported secondary cause of pouchitis, reported only in isolated cases and small series since its first description in 1998.^4^, ^5^, ^6^, ^7^, ^8^, ^9^ The largest case series describing histologically confirmed CMV pouchitis included 7 patients over a 17-year period.^6^

CMV pouchitis manifests nonspecifically, overlapping other pouch inflammation and presenting with diarrhea, fever, and abdominal pain.^6^ The gold standard for CMV pouchitis diagnosis is biopsy with microscopic identification of the characteristic CMV nuclear and/or cytoplasmic inclusions paired with positive immunohistochemistry.^10^ American Gastroenterological Association (AGA) guidelines recommend antibiotics as first-line, escalation to chronic antibiotics or immunosuppression for refractory disease, and endoscopy in atypical cases to exclude alternative etiologies.^11^

## Case Report

A 72-year-old man with a previous medical history of ulcerative pancolitis status post-TPC and IPAA complicated by chronic antibiotic-dependent pouchitis, cirrhosis secondary to primary sclerosing cholangitis (PSC), and hepatocellular carcinoma (HCC) status post orthotopic liver transplant (OLT) presented with hematochezia.

He was diagnosed with ulcerative pancolitis in his mid-50s after chronic diarrhea and rectal bleeding. He initially received infliximab, discontinued after 1 year due to nontyphoidal *Salmonella* bacteremia. His colitis progressed, and he underwent TPC with IPAA 1 year later. He subsequently developed recurrent pouchitis. Initially responsive to metronidazole, flares later required frequent antibiotics. Ustekinumab and vedolizumab were trialed but provided limited benefits. He started chronic oral vancomycin with symptomatic improvement.

One year later, he was diagnosed with HCC and subsequently underwent OLT. Post-transplant immunosuppression included tacrolimus, mycophenolate mofetil, and corticosteroids. Valganciclovir prophylaxis was stopped 3 months post-transplant per protocol.

One month after stopping CMV prophylaxis, he was hospitalized with worsening diarrhea, acute-on-chronic kidney injury, and CMV viremia (viral load 75,093 IU/mL). He was started on intravenous ganciclovir and discharged.

Two weeks later, he presented with hematochezia. Laboratory evaluation revealed hemoglobin of 7.0 g/dL (from baseline ∼10 g/dL), improving CMV viral load, and negative gastrointestinal polymerase chain reaction testing.

**Figure 1:**
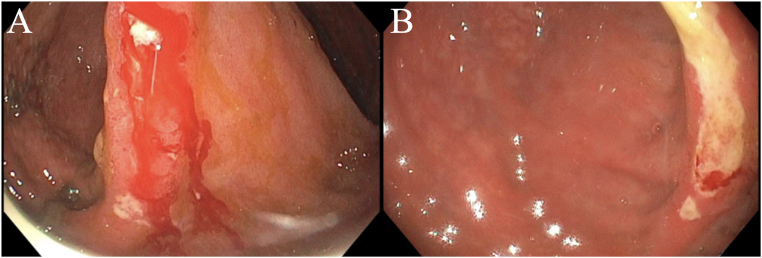
Initial pouchoscopy (A) revealing a bleeding ulcer located at the anastomosis of the pouch body and follow-up pouchoscopy (B) showing ulceration at the anastomosis of the pouch body without significant bleeding.

**Figure 2:**
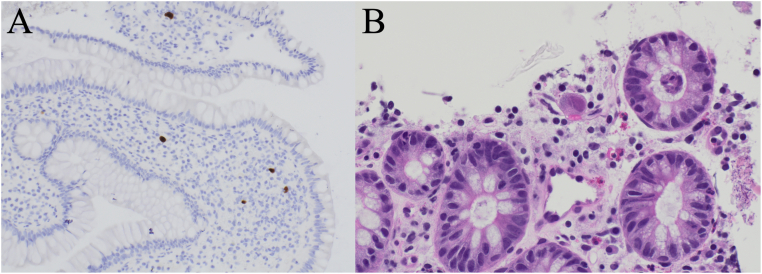
Cytomegalovirus (CMV) infection in pouch biopsy tissue. (A) Immunohistochemical staining for CMV demonstrating CMV-positive cells highlighted in brown with a hematoxylin counterstain in blue (magnification 200×). (B) Hematoxylin and eosin-stained section showing an enlarged CMV-infected cell with a prominent, glassy, eosinophilic intranuclear viral inclusion and associated cytoplasmic viral changes (magnification 600×).

Pouchoscopy revealed erythema, loss of vascular pattern, friability of the pouch body, and a bleeding ulcer in the anastomosis of the pouch body (Figure 1A). Biopsies were taken of the pouch body, pre-pouch ileum, rectal cuff, and ulcer. Hemostatic spray was applied to the bleeding ulcer. Biopsies of the pouch body and ulcer were positive by immunohistochemistry (Figure 2A) and showed CMV viral inclusions (Figure 2B).

He continued intravenous ganciclovir, transitioned to oral valganciclovir, and bleeding gradually resolved as viremia improved. Two months after his initial pouchoscopy, his CMV viral load was undetectable. However, bleeding recurred at a lower severity, occurring intermittently every several days, with his hemoglobin remaining at baseline. Follow-up pouchoscopy revealed ulceration of the pre-pouch ileum, pouch, and cuff (Figure 1B), like prior findings but without significant bleeding. Biopsies demonstrated severe active ileitis of the pre-pouch ileum and pouch with mucosal erosion and rare CMV-positive cells, while the rectal cuff showed severe active inflammation without CMV staining. Given persistent severe inflammation despite antiviral therapy and only rare CMV-positive cells on repeat biopsy, guselkumab was initiated for refractory inflammatory pouch disease.

## Discussion

This case demonstrates CMV infection as a secondary infectious complication contributing to pouch inflammation in a patient with long-standing antibiotic-dependent disease. According to AGA definitions,^11^ his course was consistent with chronic antibiotic-dependent pouchitis.

Immunosuppression was a possible factor in CMV reactivation. Our patient had several potential risk factors, most notably post-transplant immunosuppression following OLT for PSC cirrhosis complicated by HCC. In the largest reported case series of CMV pouchitis (n = 7), 71% of patients were on immunosuppressive medications, and 57% had undergone OLT for PSC, suggesting a potential association between immunosuppression and CMV pouchitis.^6^ In our patient, systemic CMV disease occurred shortly after discontinuation of valganciclovir prophylaxis. Viremia was first detected during admission for fatigue and acute kidney injury, but subsequent gastrointestinal bleeding revealed the CMV pouchitis. Although CMV viremia resolved with antiviral therapy, repeat pouch biopsies demonstrated only rare CMV-positive cells with persistent severe inflammation, suggesting an additional inflammatory component requiring escalation of therapy.

Consistent with AGA recommendations,^11^ atypical features, such as overt gastrointestinal bleeding and anemia, prompted endoscopic evaluation. This revealed ulcerated mucosa within the ileal pouch, and biopsy confirmed CMV infection by immunohistochemical staining, establishing the diagnosis.

CMV pouchitis has been described as an infectious end-organ process responsive to antiviral therapy alone,^4^, ^5^, ^6^^,^^12^ while other cases demonstrate overlapping infectious and inflammatory contributions. For example, Yanagi et al described a case in which immunosuppressive therapy was used alongside antivirals to potentially disrupt tumor necrosis factor-alpha-mediated cycles of viral propagation.^7^ CMV has been reported alongside *Clostridioides difficile* infection in pouchitis, suggesting that infectious agents may coexist in the setting of pouch inflammation, though their individual contributions to disease activity remain uncertain.^8^ In our patient, secondary CMV infection occurred in the context of prior immunosuppression, a prednisone taper completed approximately 1 month prior to presentation, and coincided with clinical deterioration, suggesting that escalation of immunosuppressive therapy alone may have been insufficient in the absence of antiviral treatment.

This case depicts the need to consider secondary infections in refractory pouch disease. It also further potentiates the unresolved question of whether CMV acts as a central driver, emerges in the setting of inflammatory susceptibility, or reflects a bidirectional process. Further studies are needed to clarify risk factors and the interplay between pouch inflammation and CMV.
